# Self-Organized Crowd Dynamics: Research on Earthquake Emergency Response Patterns of Drill-Trained Individuals Based on GIS and Multi-Agent Systems Methodology

**DOI:** 10.3390/s21041353

**Published:** 2021-02-14

**Authors:** Hai Sun, Lanling Hu, Wenchi Shou, Jun Wang

**Affiliations:** 1 College of Engineering, Ocean University of China, Qingdao 266100, China; sunhai@ouc.edu.cn (H.S.); hulanling@stu.ouc.edu.cn (L.H.); 2 Key Laboratory of Marine Environment and Ecology, Ministry of Education, Ocean University of China, Qingdao 266100, China; 3 School of Computing, Engineering and Mathematics, Western Sydney University, Parramatta, NSW 2751, Australia; 4 School of Architecture and Built Environment, Deakin University, Melbourne, VIC 3217, Australia; jun.wang1@deakin.edu.au

**Keywords:** evacuation pattern, crowd dynamics, drill-trained, panic effect, exit choice

## Abstract

Predicting evacuation patterns is useful in emergency management situations such as an earthquake. To find out how pre-trained individuals interact with one another to achieve their own goal to reach the exit as fast as possible firstly, we investigated urban people’s evacuation behavior under earthquake disaster coditions, established crowd response rules in emergencies, and described the drill strategy and exit familiarity quantitatively through a cellular automata model. By setting different exit familiarity ratios, simulation experiments under different strategies were conducted to predict people’s reactions before an emergency. The corresponding simulation results indicated that the evacuees’ training level could affect a multi-exit zone’s evacuation pattern and clearance time. Their exit choice preferences may disrupt the exit options’ balance, leading to congestion in some of the exits. Secondly, due to people’s rejection of long distances, congestion, and unfamiliar exits, some people would hesitant about the evacuation direction during the evacuation process. This hesitation would also significantly reduce the overall evacuation efficiency. Finally, taking a community in Zhuhai City, China, as an example, put forward the best urban evacuation drill strategy. The quantitative relation between exit familiar level and evacuation efficiency was obtained. The final results showed that the optimized evacuation plan could improve evacuation’s overall efficiency through the self-organization effect. These studies may have some impact on predicting crowd behavior during evacuation and designing the evacuation plan.

## 1. Introduction

**Figure 1:**
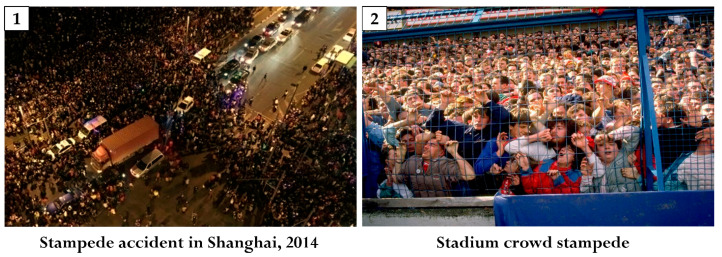
Stampede accident in Shanghai and Nepal caused by a crowd of people.

Crowd behavior in emergencies is an exciting and valuable research topic. After an urban earthquake, the demand for evacuation increases sharply in a short period. People’s behavior during an earthquake manifests as crowd behavior in emergencies. In an emergency, people tend to be crowded. For instance, on 12 March 1988, a sports competition was held at the Kathmandu Open Air Stadium in Nepal. The chaos caused by the sudden extreme weather conditions on the scene caused the audience to flee to avoid hail. The stampede of the crowd directly caused nearly 500 casualties. Not only that, another stampede accident occurred when the Muslim Hajj was held in Mecca, Saudi Arabia, on 12 January 2006, which simultaneously caused 345 deaths and nearly 400 injuries. On 31 December 2014, many people gathered at the Shanghai Bund for the New Year’s Eve countdown. Due to a large number of people at the scene and improper guidance measures, crowds colliding with each other caused some people to fall, leading directly to 36 deaths and nearly 50 injuries (see Figure 1).

It is one of the most important goals to evacuate individuals effectively in an emergency like an earthquake. However, due to the complex nature of dense urban areas and the severe lack of public emergency evacuation knowledge, unnecessary stampede injuries and deaths frequently occurred with untrained blind evacuation. Thus, the correct and efficient earthquake evacuation response of individuals could significantly reduce unnecessary casualties. Drills are a vital activity of preparedness to respond to emergencies. To effectively respond to and mitigate the impact of threats, such as the earthquake, communities must develop effective emergency strategies. Non-governmental organizations (NGOs), scholars, and government agencies have made great efforts to conduct and evaluate the effectiveness of earthquake drills. Simultaneously, some common problems were discovered, such as insufficient personnel train before the drill, and only a simple drill plan was developed to organize the earthquake emergency evacuation. It was also found that the stairs, doorways, and other areas were likely to have an immediate increase in crowd density during the drill, which were not considered in the plan [1,2,3]. However, the majority of the researches were qualitative. Most of the studies reported in the literature investigated human behaviors in earthquakes, hoping to obtain valuable solutions to provide sufficient support for evacuation decision-making [4,5]. Some of the issues being addressed are those related to finding the factors that prompt people to participate in mass evacuation drills [6]. To conduct quantitative research on the effectiveness of evacuation strategies, one of the most critical tasks is to predict people’s evacuation behavior before the earthquake. Some papers used various models to simulate the evacuation process of people in specific buildings [7,8,9,10], but there are not that many published studies explicitly considering the characteristics of people’s evacuation behavior in a panic state. It is a complicated task because the public’s awareness of disaster prevention is low, and their emergency response is various and not as expected as the same [11,12,13]. In an emergency, pedestrians may respond based on their assessment of the current environment and every possible option. Therefore, we need to establish a simulation model of the pedestrian evacuation dynamics process to obtain the optimal evacuation strategies.

Pedestrian dynamics modeling mainly includes three types, namely macroscopic model, mesoscopic model, and microscopic model [14]. The macro model interprets people’s behavior characteristics as active rather than classical particles and analyzes pedestrian movement mainly through flow, density, and speed [15]. Bellomo [16] proposed a modeling method that describes people’s movement in unbounded domains through a system of kinetic ordinary differential equations. The mesoscopic model’s object is not a single individual but multiple pedestrians in the same environment. The position and speed of the person are represented by an appropriate probability distribution. Ahmed Elaiw [17] used the kinetic theory approach to simulate two crowds moving in two opposite directions under a high-density condition including internal obstacles. They selected the kinetic theory approach to simulate crowd dynamics pedestrians in high density conditions considering both the level of stress in the crowd and the specific geometry of the areas. The micro model discusses pedestrian movement from an individual perspective and explores individual behavior and interaction. Aylaj et al. [18] developed a unified framework to derive models at each scale, which could simulate crowd dynamics in complex environments composed of interconnected areas. And this model considers the emotional state of pedestrians. The methods proposed in these papers can accurately simulate people’s rational and irrational behaviors, but these behaviors are all caused by the surrounding environment, and they do not take into account the individual’s cognitive level, psychological state, and familiarity of the environment. In summary, large-scale models often ignore the diversity of individual actions, and it is difficult to simulate all relevant behavior characteristics in the crowds, such as panic effect and exit choice. So we mainly focused on individual behaviors at the micro level and hoped to establish a model that can reflect the crowd interaction process in the evacuation process.

Several researchers used quantitative models to simulate crowd behaviors [19,20]. Among them, the social force model is the most popular model describing people’s evacuating behaviors. Helbing and Molnar [21] in 1995 firstly proposed the social force model. It is suggested that pedestrians’ motion in the evacuation process could be described as if they would be subject to “social forces.” In recent years, many scholars have improved the primitive social force model. Parisi et al. [22] introduced a “self-stop” mechanism to prevent other pedestrians from pushing the current pedestrian. Saboia et al. [23]. applied the mobile grid to the social force model to allow the simulated pedestrians to change their desired velocity direction at reasonable times. Zanlungo and Francesco [24] introduced a new social specification in which applied genetic algorithms to predict the next collision’s place and time. Hou at al. [25] proposed an improved social force (SF) model. Their simulation results indicate that the trained leader’s proper guidance would be an essential method to accelerate the dynamic, especially when the visibility range is limited.

On the other hand, an animal experiment using ants as study objects conducted in 2005 intended to show that individuals use two exits non-symmetrically under panic conditions, but this phenomenon did not happen normally. It reflected that panic could influence their rational thinking. Helbing et al. [26]. used a model of pedestrian behavior to analyze the mechanisms of panic. It proposed an optimal strategy to escape from a smoke-filled room, involving a mixture of individualistic behavior and ‘herding’ instinct. Considering the influence of personnel’s mental state on panic behavior, Moch et al. [27] believed that the person most familiar with the building environment should be appointed as the person in charge and brought others to a safe place. Cheng [28] endowed multi-agent simulations with environmental consciousness, flight route planning ability, and information interaction ability, and plans the ocean platform’s escape route based on the A* algorithm. The research mentioned above has gradually considered human behavior under the influence of subjective and objective factors. The research combining the panic mental state of personnel and considering both the macro and micro levels of personnel behavior is still lacking. Most studies mainly focused on the distance to the exit, the degree of congestion, the exit’s width, internal obstacles, and other factors. Some studies mentioned the panic effect and export familiarity’s impact did not conduct quantitative assessments [29,30].

Currently, we found some exciting phenomenon in emergent conditions through empirical methods. Spending more time and cost to prompt the general public to participate in mass evacuation drills would not achieve good evacuation effectiveness. For example, it takes less time to cut corners, which can effectively avoid congestion. However, as more and more people take shortcuts, shortcuts would become more congested. The ideal result of evacuation is that multiple exits could be reasonably and fully utilized, and people could reduce the evacuation time by continuously reducing the distance with others. So far, there are not that many published studies addressing how to organize scientifically to improve mass evacuation drills’ effectiveness.

This paper intends to address this gap using a computer-aided quantitative simulation. Here an evacuation drill experiment was designed and conducted to analyze people’s actual evacuation process under emergency occasions, especially personnel participation ratio and evacuation behavior characteristics. To better describe and simulate people’s evacuation process, some improvement measures were made to describe people’s behavior quantitatively in emergencies: (1) Established the response rule equation of the crowd in emergencies. (2) Quantitative description of panic behavior and exit familiarity. (3) Quantified the relationship between exit familiarity and drill training time. Based on the above models, we conducted a simulation based on the cellular automaton. We simulated different exercise strategies by setting different export familiarity ratios. The experiment was intended to optimize the evacuation process’s efficiency through the self-organization of personnel and avoid unnecessary congestion and stampede accidents. The results showed that the exit familiarity caused by different drill strategies might disrupt the balance of their exit choices, causing some of them to be crowded. And due to people’s rejection of long distances, congestion, and unfamiliar exits, some people would hesitant about the evacuation direction during the evacuation process. This hesitation would also significantly reduce the overall evacuation efficiency. Apparently, in many cases, the crowd’s irrational behavior with panic emotion in emergencies could result in unfortunate consequences. However, we could also utilize this effect to optimize our existing evacuation strategy. Finally, we took a community in Zhuhai City, China as an example, and put forward the best disaster prevention drill strategy.

## 2. Model Construction

Many models could describe people’s evacuation behaviors, among which the continuum model, floor field model, and cost potential field model are popular based on the cellular automaton model. Many researchers have proved that these models could simulate people’s evacuating process well and developed valuable findings and advice. While most of the present simulation models merely mentioned the influence of panic effect and exit familiarity, both had not quantitative studied these factors’ effects. These models could not correctly simulate when people are quite familiar with exit and emergency. To better describe and simulate people’s evacuation process, some modification was put forward based on the above phenomena that we have introduced in detail.

### 2.1. General Description of the Integrated Model

We have chosen a continuum model for basic simulation. A continuous simulation’s benefit is that infinitesimal movements are possible and show people’s movement more naturally. The continuum model is mathematically described as:

(1) $$m\frac{\delta^{2}x_{p}}{\delta t^{2}}=-\overset{N}{\underset{q=1,q\neq p}{\sum }}\nabla_{x_{p}}V_{agent}\left(\left|x_{p}-x_{q}\right|\right)-\nabla_{x_{p}}V_{exit}\left(\left|x_{p}-x_{q}\right|\right)-\overset{W}{\underset{q=1}{\sum }}\nabla_{x_{p}}V_{boundary}\left(\left|x_{p}-x_{q}\right|\right)$$

**Figure 2:**
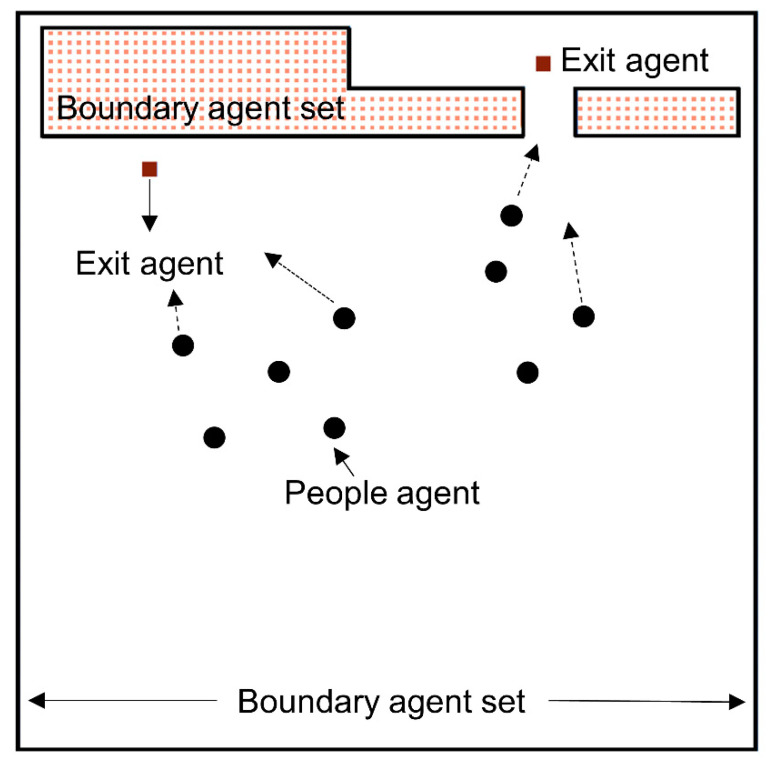
Schematic diagram of the constituent elements of the continuum model.

The elements are shown in Figure 2, where $V_{agent}$ represents potential-field of other individuals, $V_{wall\;}$ represents potential-field of the boundary, $V_{door}$ represents potential-field of the exit which an individual agent is heading for.

Since the boundary and exit elements could not move, they build a static field together. We constructed the boundaries using a row of fixed agents. Considering the natural behavior of avoiding walking close to the boundary, we used repulsive boundary potentials inversely proportional to the boundaries’ distance:

(2) $$V_{boundary}\left(r\right)=k_{W}\frac{1}{r}$$

The range of the boundary effect is limited to the maximum distance $D_{max}$ from the boundaries. This parameter avoids considering the wall on the other side of the room. $k_{w}$ represents a constant, describes how strong the repulsive force of the boundary is.

The active part of the field comes from moving individuals. Everyone in the room creates a field that excludes the other agents. Therefore, this has a significant impact on how people move in the area. The following equation expresses it. We used in our simulation that the agents have another constant $k_{A}$ in front of the 1/r:

(3) $$V_{agent}\left(r\right)=k_{A}\frac{1}{r}$$

The exit potentials are almost similar to the boundary potential. The most significant difference here is that they are attractive. The force is proportional to the square of the distance which an agent is away from. $k_{D}$ is a constant which represents the strength of the attracting force caused by the exit. An s factor is also required since the potential would have a zero gradient if the radius is zero:

(4) $$V_{\mathrm{exit}}\left(r\right)=k_{D}{\left(r+s\right)}^{2}$$

The exit and boundary elements are formed by a row of agents that are uniformly distributed. This equation allows us to simulate realistic escape dynamics. All different potentials result in a single force acting on the agent. According to this field, the individual agent reacts and moves.

### 2.2. Exit Choice Process

In an emergency evacuation, the selection of the exit is one of the most critical decisions. Previous research found that people usually choose their exit rationally based on their evaluation of evacuation time, queuing time, exit widths, and anticipated congestion level. We considered that agents would choose the fastest evacuation route. Despite the time-related factor, we included two factors: familiarity and visibility of the exits. An agent’s estimated evacuation time is the sum of the estimated moving time and the estimated queueing time. The estimated traveling time is estimated by dividing the distance to the exit by the agent’s velocity. The estimated queuing time needs to consider the exit’s capacity and the number of the other nearer agents heading towards the exit. The estimated queuing time links the decision of a single agent with the others. In short, this means the fastest exit choice for a particular individual may change during the evacuation.

The agents are identified with indices i and j, where i,j∈N={1,2,3,…,N}. Exits are denoted by $e_{k},k\in K=\left\{1,2,\ldots ,K\right\}$. Strategies are denoted by $s_{i}\in \left\{e_{1},\ldots ,e_{K}\right\}=S_{i},i\in N$ where $S_{i}$ is a strategy set of agent i.

The estimated move-time depends on the position $r_{i}$ of agent i and the position $b_{k}$ of exit $e_{k}$. The location of the agents is in the collection $r:=\left(r_{1},\ldots ,r_{N}\right)$. Therefore, the distance between agent i and the exit c is:

(5) $$d\left(e_{k},r_{i}\right)=∥r_{i}-b_{k}∥$$

The estimated traveling time of agent *i* is the division of the distance $d\left(e_{k};r_{i}\right)$ by agent *i*’s velocity $v_{i}^{0}$:

(6) $$\tau_{i}\left(e_{k},r_{i}\right)=\frac{1}{v_{i}^{0}}d\left(e_{k},r_{i}\right)$$

The estimated queueing time is calculated by the sum of all agents except agent i traveling towards exit $e_{k}$, which is closer to exit $e_{k}$ divided by the exit $e_{k}$’s capacity $\beta_{k}$.

The subset of all agents i≠j who are closer to $e_{k}$ than agent i is given by:

(7) $$\Lambda_{i}\left(e_{k}\right)=\left\{j\neq i|s_{j}=e_{k},d\left(e_{k},r_{j}\right)\leq d\left(e_{k},r_{i}\right)\right\}$$

The number of elements in the collection $\Lambda_{i}\left(e_{k}\right)$ is denoted by:

(8) $$\lambda_{i}\left(e_{k}\right)=\left|\Lambda_{i}\left(e_{k}\right)\right|$$

The capacity $\beta_{k}$ of exit $e_{k}$ is a scalar value that means how many agents can pass the exit $e_{k}$ at one time, so the estimated queueing time is:

(9) $$\frac{1}{\beta_{k}}\lambda_{i}\left(e_{k}\right)=\frac{1}{\beta_{k}}\left|\Lambda_{i}\left(e_{k}\right)\right|$$

The estimated evacuation time is sum of the estimated moving time and estimated queueing time of agent i through the exit $e_{k}$:

(10) $$T_{i}\left(e_{k}\right)=\frac{1}{\beta_{k}}\lambda_{i}\left(e_{k}\right)+\tau_{i}\left(e_{k},r_{i}\right)$$

As a result of the principle of the game theory, every agent would choose the exit which has the shortest evacuation time:

(11) $$s_{i}=\underset{e_{k}\in S_{i}}{min}T_{i}\left(e_{k}\right)$$

**Figure 3:**
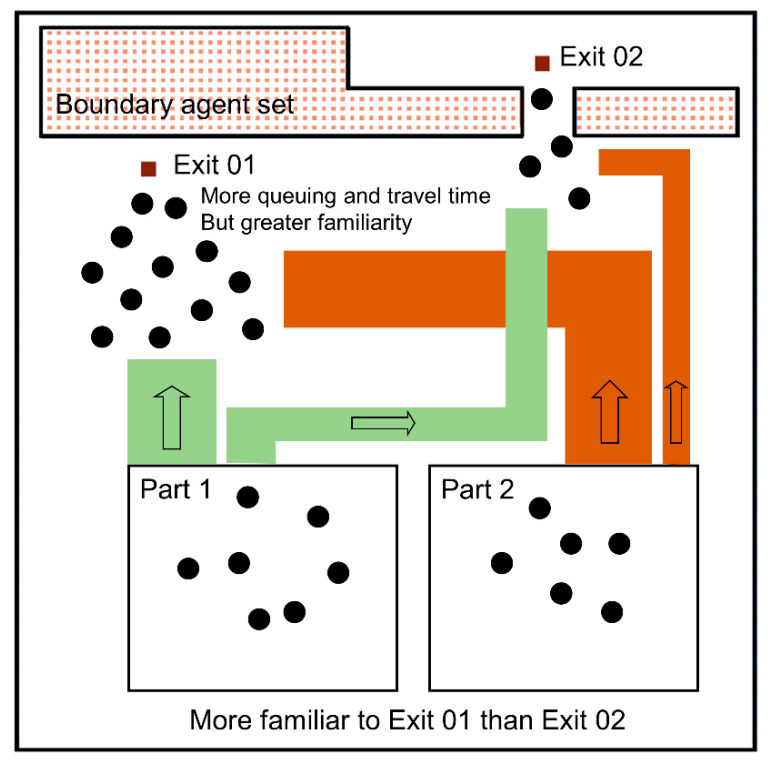
Exit choices for people in different places.

The familiarity and visibility factor limit the possible exits. These factors could be seen as binary signs, and the number of possible combinations constitutes a preference group. Agents would choose an exit from the non-empty group with the highest priority, as shown in Figure 3. The phenomenon that people would like to choose their familiar exit to escape also exists on many other occasions. It is called the exposure effect or the familiarity principle in psychology. For example, people would like to make friends with people they frequently see, and investors would change their risk perception according to their familiarity with risk events when making investments.

**Figure 4:**
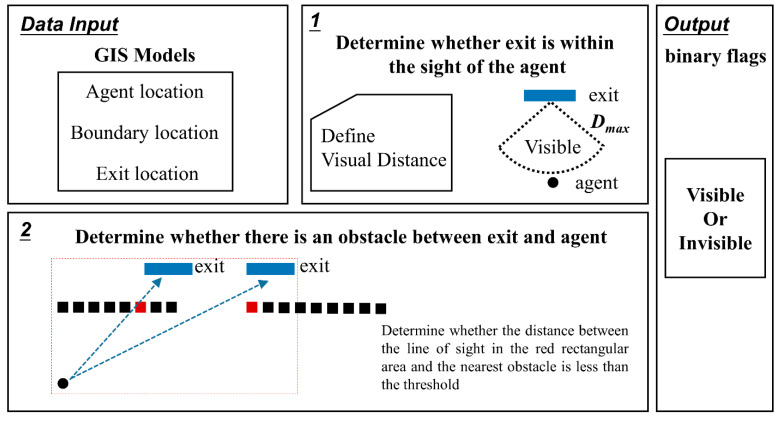
The methodology to judge whether the exit is visible to the agent.

As mentioned before, the impact of familiarity and visibility of exits may limit the set of possible exits for agent i, and these conditions are considered by defining two binary flags $fam_{i}\left(e_{k}\right),vis\left(e_{k},r_{i}\right),\forall i\in N,k\in K$. The step to judge whether the exit $e_{k}$ is visible to agent i is showed in Figure 4. The binary flags give specific information about agent i:

(12) $$fam{\;}_{i}\left(e_{k}\right)=\{\begin{array}{cc} 1 & if\;exit\;e_{k}\;is\;familiar\;to\;agent\;i\;\\ 0 & if\;exit\;e_{k}\;is\;not\;familiar\;to\;agent\;i \end{array}$$

(13) $$vis\left(e_{k},r_{i}\right)=\{\begin{array}{cc} 1 & \;if\;exit\;e_{k}\;is\;visible\;to\;agent\;i\;\\ 0 & if\;exit\;e_{k}\;is\;not\;visible\;to\;agent\;i \end{array}$$

There are four possible combinations: four groups of exits with priority numbers from 1 to 4. The lower the priority number is, the more desirable the exit. The familiarity of an exit has a more significant influence on how preferable an exit is. Research shows that evacuees will choose a familiar route, even the route is short. The visibility flag is vital for calculating the estimated queueing time because an agent can only estimate the queue in front of an exit if he can see the exit. Figure 4 could also reflect relevant information. We grouped the exits, as shown in Table 1.

**Figure 5:**
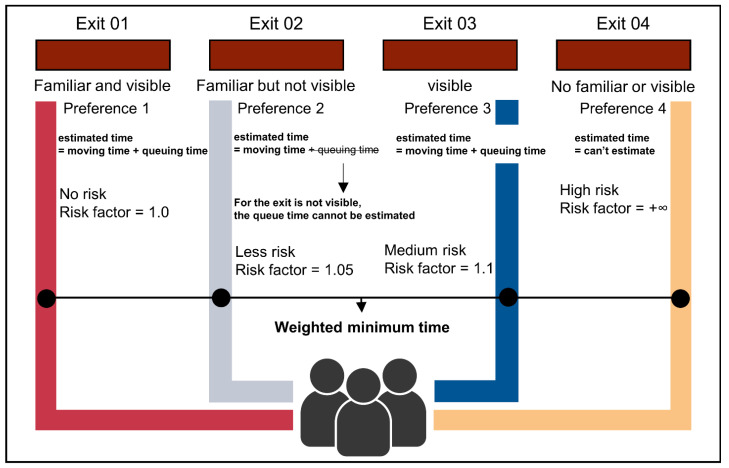
Decision-making process of an agent.

The fourth preference group describes people in a panic who are not familiar with the exits and could not see any exit. Mathematically speaking, the choice of the exit is defined as $s_{i}=\underset{e_{k}\in S_{i}}{min}T_{i}\left(e_{k}\right)$ and $s_{i}\in E_{i}\left(\overset{-}{z}\right)$, The specific agent i chooses an exit from the non-empty group $E_{i}\left(\overset{-}{z}\right)$, which has the best priority number $\overset{-}{z}$. In addition to this paper, we have also added an extra risk factor. The risk factor is a simple comparison between the preferable new exit’s evacuation time and the previously chosen exit. This step is needed because it may happen that the exit in a better preference group the evacuation time could take much longer, and a better exit gets insight. So, the agent would redecide if the new preferable exit’s evacuation time is greater than the evacuation time of the agent’s previous decision. Nevertheless, to choose an unfamiliar or invisible exit, the agent may face more risks. Therefore, a risk factor was defined to quantify the decision process’s time risk, as shown in Table 2. Finally, Figure 5 briefly illustrates this process.

### 2.3. Quantitative Description of Panic Behavior and Drill-Trained Effect

#### 2.3.1. Panic Behavior 

According to previous theories, there was an irrational phenomenon of panic behavior that people would like to imitate others’ behavior. We defined people in a panic who is not familiar with the exits and could not see any.

**Figure 6:**
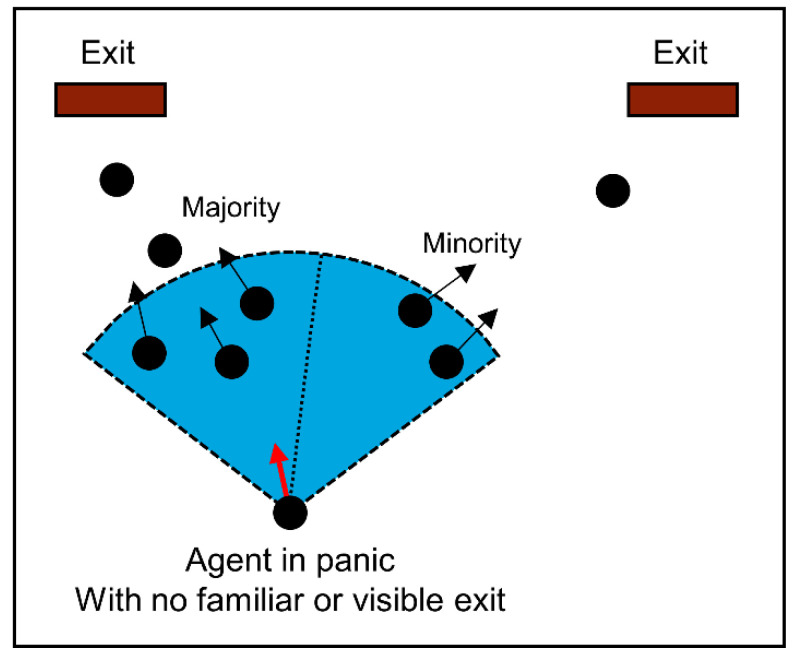
Panic behavior of the agent with no familiar and visible exit.

We assumed that the direction with larger crowd density and velocity has larger attraction to the individual under the nervous psychological condition for the panic behavior term, see Figure 6, so we could define an attractive potential term caused by a panic effect, which satisfies:

For agent i, who is in a panic: $V_{panic\;agent}\left(i\right)=\frac{1}{n}\sum_{d\left(i,j\right)<Dvis}V_{j}$, and the set of agent j are both toward the most crowded exit.

The range of the attractive potential effect is also restricted up to the distance $D_{vis}$ from the people agent. This parameter represents people’s vision in panic situations. $V_{panic\;agent}\left(i\right)$ represents the speed of agent i, n represents the number of agent j within the range of distance less than $D_{vis}$.

#### 2.3.2. Drill-Trained Effect 

**Figure 7:**
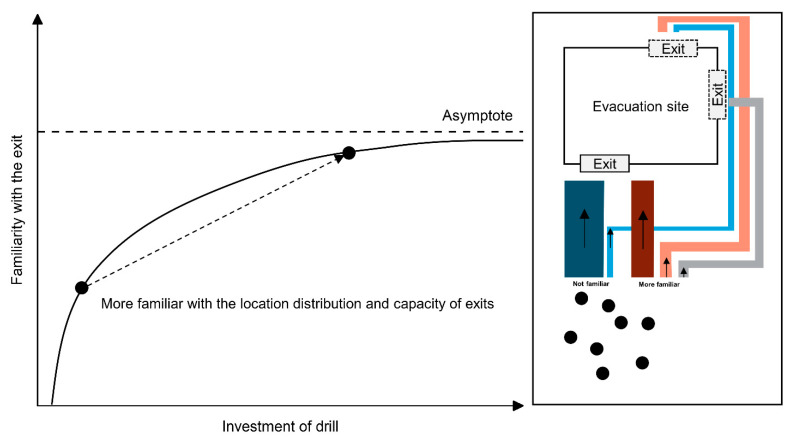
Lally’s habit formation model and the effect of the drill.

As for the drill effect, we defined a familiarity ratio *ξ* (0 ≤ *ξ* ≤ 1) based on Lally’s habit formation model:

(14) $$\xi_{0}=a-be^{-cT}$$

where $\xi_{0}$ represents people’s habit formation level, *T* is the time length starting from the training time, a, b and c are constants. Among them, a represents the asymptotic value, b controls the starting time, and *c* controls the habit formation speed. People would be more familiar with each exit’s distribution and capacity, and the familiar proportion changed accordingly, as shown in Figure 7.

## 3. Original Model Simulation

**Figure 8:**
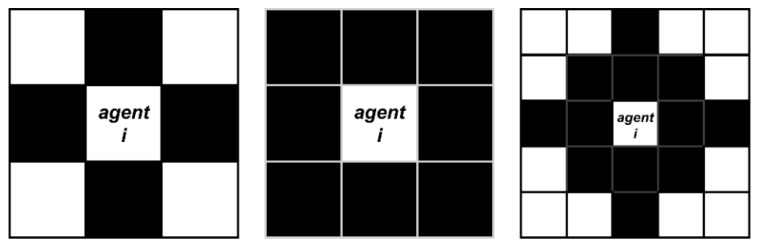
Definition of cellular automaton neighborhood range. (**a**) Von Neumann; (**b**) Morre; (**c**) Extensional Moore.

A small simulation based on a cellular automaton was conducted to explain the modification to the integrated model further. A 40 × 40 rectangle with two exits located at the two ends of the rectangle was used to simulate the evacuation drill’s crowd evacuation process. One of the exits was in a prominent position to represent the exit that most people are familiar with. The other exit was located in a relatively inconspicuous position, whose actual capacity was also low. Therefore, most people were not familiar with it, for it was not easy to cause people’s attention. The length of 1 unit in this simulation represents 5 m in reality. One hundred fifty people were randomly located in a fixed area for each simulation. For each time interval, the people agent could only move to his adjacent eight cells, see Figure 8. At the beginning of the simulation experiment, each agent randomly set the preferred and familiar exit. Simultaneously, the initial speed was also set.

During the simulation, each individual decided his moving route by the potential. The potential was decided by the sum of the forces of other types of agents. The factor of exit familiarity and panic effect were both added in the simulation.

**Figure 9:**
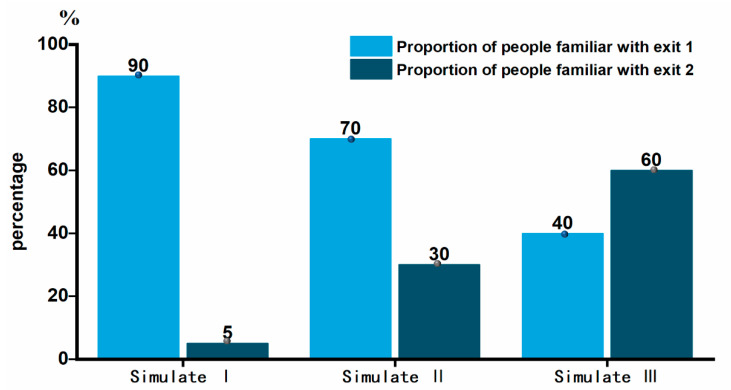
Exit familiar ratio for people in different simulations.

In simulation I, which represented the original circumstance, the crowd only received the basic drill education roughly and probably know the primary exit location and had not undergone drill training. For this circumstance, 90% of the individuals were set to be more familiar with Exit 1, only 5% of the individuals were set to be familiar with Exit 2. For the second circumstance, 70% of the individuals were set to be more familiar with Exit 1, and 30% of the individuals were set to be familiar with Exit 2, and this ratio setting was decided by the total number and time of drills they received. The third circumstance considered more people choose to take Exit 2 because they had received sufficient drill training. The proportion of evacuation from Exit 1 and Exit 2 was set to 40% and 60%, respectively. The familiar ratio of the grouped simulation was shown in Figure 9.

The simulation was conducted using the integrated model in a simple evacuation scenario, including the familiarity and panic effect. Here we chose the corresponding parameters as $W_{eps}\;$= 0.1, which represents the spacing between boundaries, people $R_{ad}$ = 0.7, which indicates that the allowable radius of a person agent is 1. $V_{max}$ means that the maximum speed of a person is 10. $K_{A}$ in Equation (3) is equal to −60, which is the same as the exit’s attractiveness, reflecting that people may stay still if there is congestion at the exit. Additionally, $K_{wall}$ = −5, $K_{exit}$ = −60, respectively.

**Figure 10:**
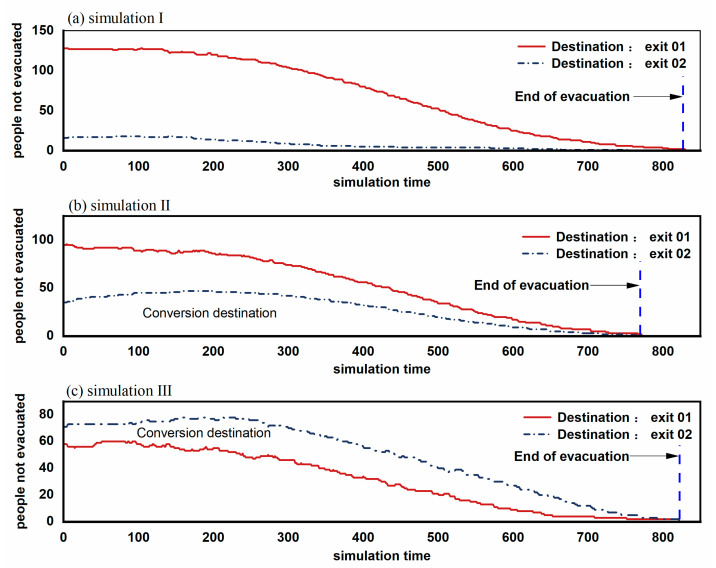
Numbers of moving people with different destinations during the evacuation.

The total simulation results were shown in Figure 10, where we could see the number of people not evacuated versus the time. In simulation I, the total evacuation time was 813 steps, which shows no significant difference from the third circumstance. In contrast, the second circumstance consumed about 760 steps and declined by about 7% in time, showing the highest evacuation efficiency.

**Figure 11:**
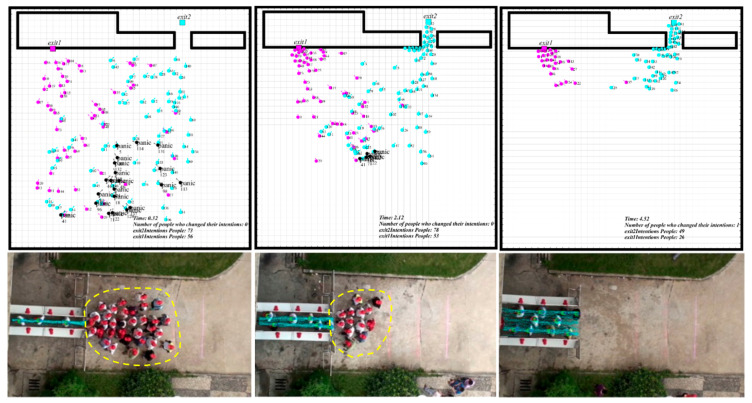
The crowd evacuation process in simulation I [31].

In simulation I, the number of people evacuating from Exit 1 and Exit 2 was 132 and 18. Results show that exit familiarity could cause the two evacuating exit choice disequilibrium, which results in the congestion in one of the exits. In comparison, the low evacuation efficiency caused by an imbalanced population at two exits could also be found on this occasion, see Figure 11. The congestion effect generated in the simulation process fitted well with the real experiment [31].

In simulation II, the number of people evacuating from Exit 1 and Exit 2 was 93 and 47. With time going by, the distances between nearby individuals got closer and closer. People’s flow at the two exits was relatively even, and there was no prominent congestion area. One possible reason was that the distribution ratio of the people was consistent with the exit capacity approximately. It is an ideal result for an evacuation to reasonably and fully utilize all of the exits. People could reduce their evacuation time by continuously decreasing the distances to other people. Simultaneously, more people were familiar with both of the two exits, more people might make reasonable changes to the route by judging the time during the evacuation. People’s distances heading for the same exit got close again. This phenomenon indicates that optimizing each individual’s exit choice would simultaneously optimize the overall evacuation process and raise people’s evacuation speed. It should also be noted that the premise of exit choice optimization was that people should be familiar with each exit before.

**Figure 12:**
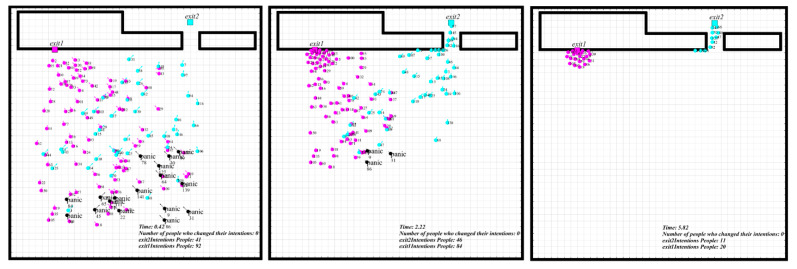
The crowd evacuation process in simulation II.

**Figure 13:**
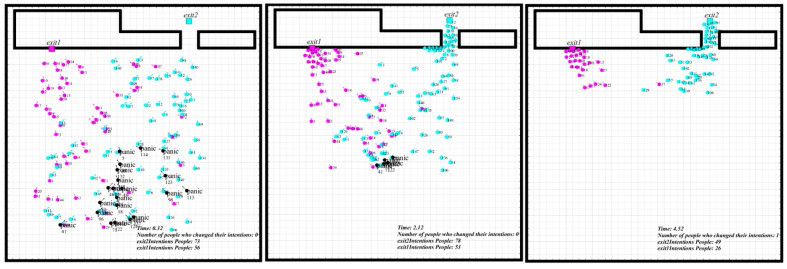
The crowd evacuation process in simulation III.

The third circumstance increased people’s familiarity ratio with Exit 2 based on the second circumstance. The total evacuation time showed no significant change than the first circumstance, while the evacuation process was quite different. For simulation 3, part of the individuals was indecisive for the evacuating direction during the evacuation. One of the reasons was that Exit 2 was relatively hidden, and it was not visible to many people. Although more people were familiar with Exit 2 than simulation 2, they could not assess the time spent in line because the exit was not visible. They were trapped by the hard choice of long-distance, high congestion level, and less familiar exit, and this kind of hesitation could significantly reduce the whole evacuation speed. We also found the lower evacuation efficiency caused by the imbalanced congestion and indecision from the comparison of Figure 12 and Figure 13 at the same time.

We had obtained two necessary conclusions through the above simulation process: Improving people’s familiarity with the exit through the drill could speed up the evacuation process and improve the evacuation efficiency. The second was that optimizing the proportion of the exit familiarity need comprehensively consider factors such as export capacity and degree of concealment.

## 4. Case Study via Developed Model

This paper took a realistic earthquake case as an example to validate the improved continuum model used in an urban evacuation scenario.

### 4.1. Study Area and the Layout of the Community

**Figure 14:**
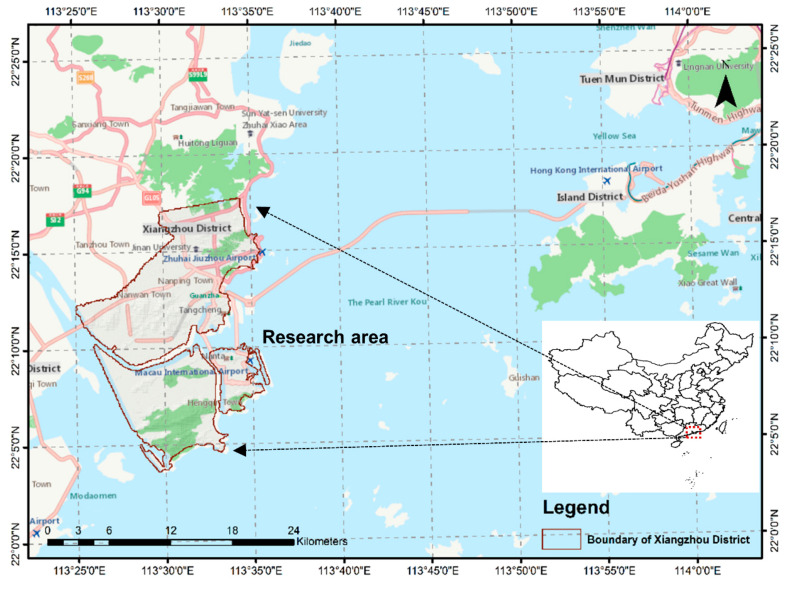
Location of the case study area, Xiangzhou District, Zhuhai.

The realistic experiment was conducted in the Huaping community, Xiangzhou District, Zhuhai City. Zhuhai City is one of the Pearl River Delta’s central cities and an important node city in the Guangdong-Hong Kong-Macao Greater Bay Area. Zhuhai City is located in the south-central part of Guangdong Province, facing Hong Kong and Shenzhen across the sea, and connected to Macau, see Figure 14. Its geographical position is exceedingly significant. Zhuhai City covers 1711.24 square kilometers and has three administrative districts: Xiangzhou District, Jinwan District, and Doumen District. Xiangzhou District is the capital city of Zhuhai, Guangdong Province. It is located between 21°48′ N to 22°27′ N and 113°32′ E to 114°18′ E. The administrative area is 555.29 square kilometers. The territory’s total permanent population is 937,800, including 647,100 registered population at the end of 2018.

At 6:55 am on 5 January 2020, a magnitude 3.5 quake occurred in the sea area of Xiangzhou District, Zhuhai City, with a focal depth of 12 km. The quake had a wide range and was prone to causing secondary disasters.

**Figure 15:**
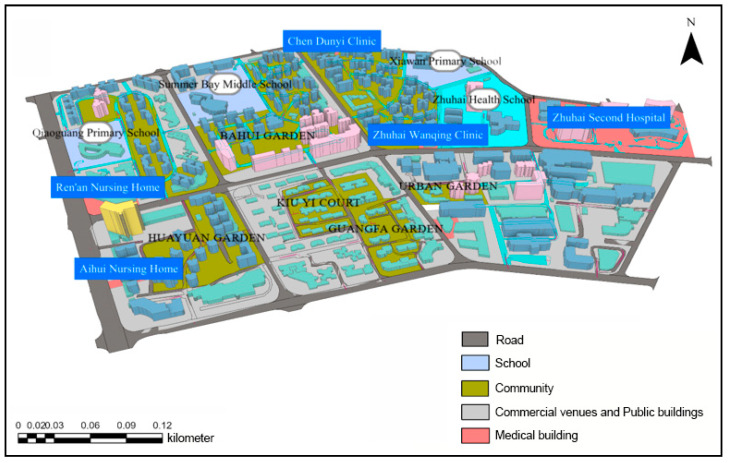
Functional zoning map of Huaping community and surrounding areas.

Huaping Community and its surrounding areas are located in Gongbei Street, Xiangzhou District, Zhuhai City, Guangdong Province. It is enclosed by Gangchang Road, Xiawan Road, Guihua South Road, and Qiaoguang Road, covering an area of 917,670 square meters and has a total of 288 buildings and a population of 19,948. There are eight residential communities, three elementary and secondary schools, one technical secondary school, two kindergartens, five medical institutions, and more than two large-scale living quarters. We selected this area as the research object (see Figure 15), considering a complete transportation network, different living facilities, and reasonable regional planning.

At the instance of the relevant disaster prevention principles: medical treatment and safety should be met by emergency shelters; 14 locations were selected as candidate emergency shelters. The effective shelter area per capita should be equal to or greater than 1.5 m^2^. Therefore, the area of emergency shelters selected and the capacity of evacuees were as following (see Table 3).

### 4.2. Data Preprocessing

**Figure 16:**
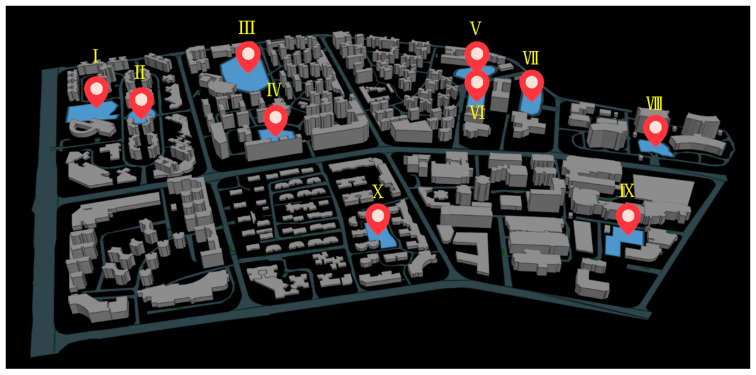
Visualized environment for emergency evacuation in the study area.

This paper used GIS to establish a three-dimensional emergency evacuation environment and obtained the optimal route through OD cost analysis. Scenario simulation was visualized by Multi-Agent Systems (MAS) technology in the above environment, see Figure 16.

OD cost analysis could realize path cost calculation from multiple starting points to multiple destinations. The optimal route based on OD cost analysis is the prerequisite for determining the location of emergency shelters. The determining factors of the community emergency evacuation network, which include the level of danger of roads and the pedestrians’ velocity based on OD cost analysis. Under the influence of different magnitudes, buildings have varying degrees of damage and destruction. In severe instances, the accessibility of roads would be affected by the collapsed buildings. When there are too many collapsed buildings around, the road’s risk would be higher, and the reliability would be lower. Then a higher reliability coefficient road would be selected. Assuming that there are k buildings on a specific route, the number of buildings that collapse and affect the route is n, and the probability of each building collapse is p, then the reliability coefficient is $R=1-C_{k}^{n}p^{n}{\left(1-p\right)}^{k-n}$.

The movement speed of ordinary people is 1.5 m/s. Considering the people being affected by panic, the standard escape speed on the main road is 1.35 m/s. The escape velocity would be disturbed by building collapse. Adopting the standard speed $V_{0}$ and the reliability coefficient R, the modified escape speed is $V=V_{0}\times R$. We saw each route’s evacuation time as an essential index for evaluating the best escape route.

### 4.3. Simulations and Result

The parameters were set as following: the radius of each pedestrian, r = 0.3 m, the average pedestrian’s desired velocity was set to a normal distribution with a mean of 1.35 m/s. Two hundred eighty-eight agent generators (from building exits) and ten agent collectors (shelters) were established in the simulated scenario. The evacuation routes of the pedestrians in the early stage were put into the emergency evacuation visual environment.

**Figure 17:**
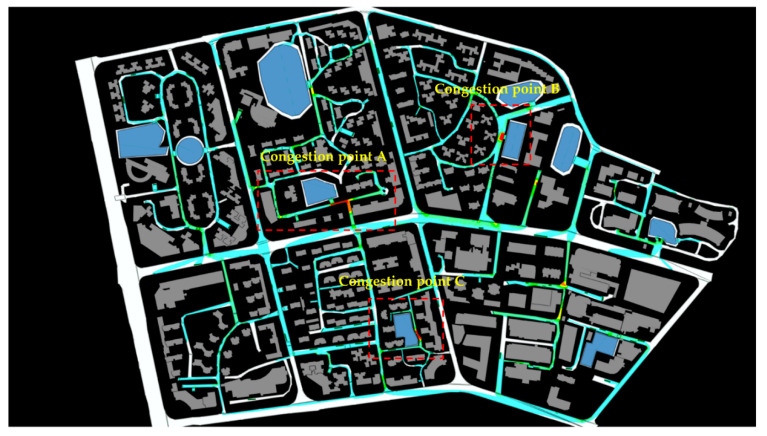
Maximum density map of the whole area in case01.

**Figure 18:**
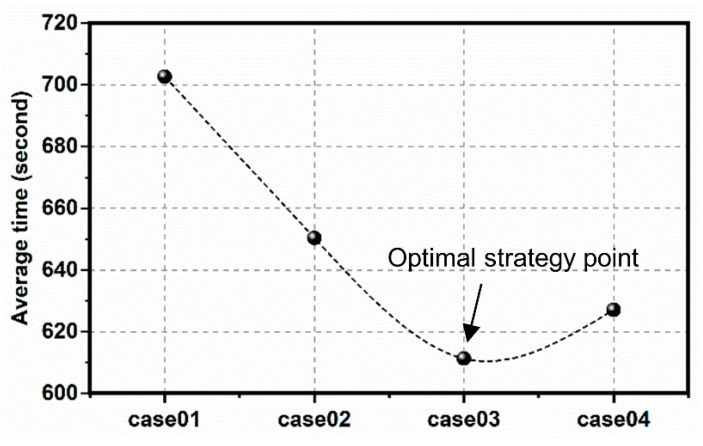
Time chart of receiving agent in each emergency shelter.

**Figure 19:**
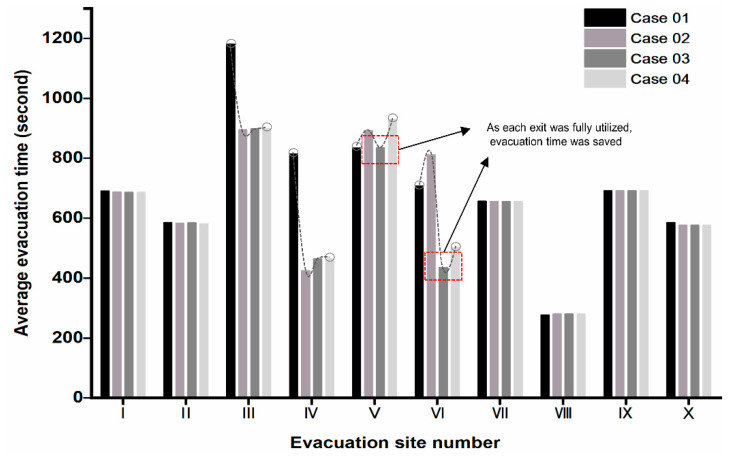
The average evacuation time of different cases.

For the pedestrians, we defined the exit planned in the early evacuation route as the optimization exit, the exit around the optimal exit as the opportunity exit. For instance, the pedestrian familiar with the exits should know all the designated shelter’s entryways. We set case 01 as a benchmark group in which pedestrians had no preparation and plan, with 5% familiarity with the optimization exit and 5% familiarity with the opportunity exit. We continuously increased the proportion of opportunity exit familiarity of pedestrians, setting 5%, 10%, and 15% opportunity export familiarity ratios in case 02, case 03 and case 04. The impact of familiarity on the evacuation time was analyzed, and results were shown in Figure 17, Figure 18 and Figure 19.

#### 4.3.1. Drill-Trained Effect

The evacuees’ training level could affect the evacuation pattern and clearance time of a multi-exit zone.

The entire simulation time of case 01 was 19 min 42 s, and the average evacuation time was 11 min 7 s. There was still a sizeable accommodating space in the shelter I and III showing that the resources were not effectively utilized. The possible reason may be that pedestrians were not familiar with shelters I and III. The end time of each evacuation site varied greatly among emergency shelters. It was necessary to conduct drill planning to improve the balance of the use of shelters.

In case 02, we assumed that the pedestrians were educated but not been drilled, with 95% optimization exit familiarity and 5% opportunity exit familiarity. The overall evacuation efficiency was much improved. The emergency shelters’ evacuation time was 14 min 56 s, shortened by 4 min 46 s. The average evacuation time was 10 min 56 s, shortened by 12 s compared to the initial deduction’s initial average time. At the same time, all emergency shelters did not exceed the capacity limit. All evacuees completed the evacuation tasks within the stipulated time. Each emergency shelter’s completion time was slightly reduced, such as Ι, ΙΙ, VΙΙ, and X, while V, VΙ, VΙΙΙ, and ΙX increased. Estimating from the alteration in the region’s maximum congest density map, case 02 effectively relieved the congestion along the road leading to refuge IV. However, it did not alleviate the congestion of the other two divisions.

In case 03, we assumed that the pedestrian had been moderate drill-trained, with 95% optimization exit familiarity and 8% opportunity exit familiarity. The average evacuation time was 10 min 28 s, 29 s shorter than case 02, and the overall evacuation efficiency was significantly ameliorated. The evacuation time of refuge VΙ was 7 min 16 s, 7 min less than the case02’s evacuation time. The evacuation time of V was 13 min 56 s, 1 min 34 s less than case 02. The evacuation time of other emergency shelters remained unchanged. Under the maximum density map observation, case 03 effectively alleviated the road congestion around refuge IV and VI. It relieved the intersection congestion around refuge X. The local density of these refuge areas no longer exceeded 5 ppl/m^2^. It is an ideal solution for an evacuation that all exits are reasonably and fully utilized. People reduce their evacuation time by continually decreasing the distance to others.

In case 04, we assumed that the pedestrians were well-trained, with 95% optimization exit familiarity and 15% opportunity exit familiarity. Compared with case 03, the overall evacuation efficiency of case 04 had not been ameliorated. Furthermore, the evacuation completion time of VΙ is 8 min 23 s, which was 1 min 7 s longer than the previous case’s evacuation time. From the perspective of the changes in the maximum density map of the congested area, the pedestrian’s increased opportunity exit familiarity had no significant effect on alleviating the entire road network’s congestion.

Opportunity exit familiarity had a positive impact on the Huaping community’s evacuation. As the ratio of this group of people increased, the overall evacuation efficiency firstly increased and then decreased, see Figure 18. Therefore, it is necessary to control the proportion of this group of people.

#### 4.3.2. The Influence of People’s Exit-Choice Decision

**Figure 20:**
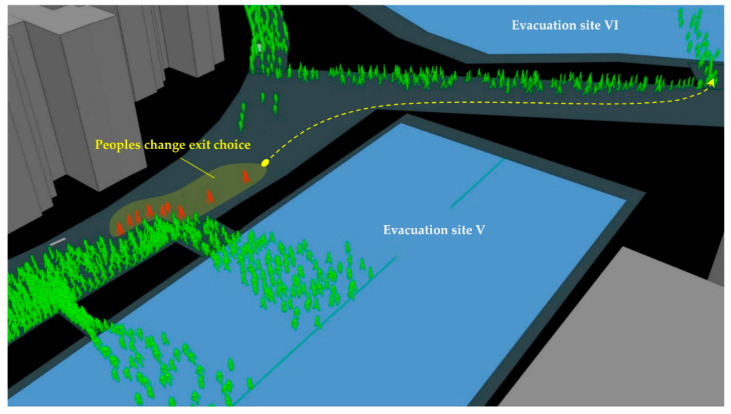
Schematic diagram of the panic effect of evacuation simulation.

**Figure 21:**
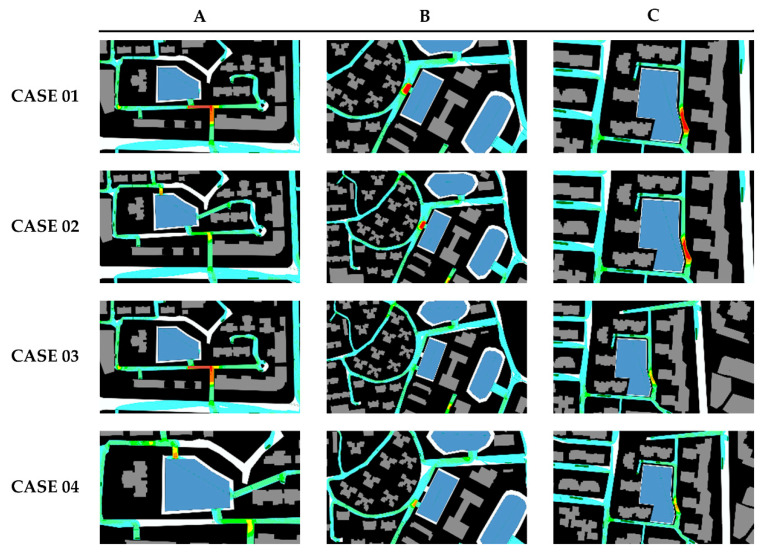
Maximum density map of congested areas in simulations.

The dynamic interaction of people concerning the exits’ congestion state may influence the process of the evacuation. Due to the long distance of the target and the unclear vision, some pedestrians were hesitant about the evacuation direction during the evacuation process. This phenomenon will significantly shorten the overall evacuation speed and lead to exit congestion. We could observe the decision-making process in front of the congested exit in Figure 20. Agents marked in red who are familiar with the exit but cannot see the exit firstly went to exit VI and then chose exit V. This is because the evacuees’ primary exit selection criterion was the expected travel time, which depends on the distance from the current location to the exit. When encountering severe congestion in front of the familiar exit, the criterion changed to the sum of travel time and queue time. Simultaneously, according to the improved continuum model, people who are neither familiar with the exit nor can they see the exit will cause panic and make unreasonable judgments and behaviors. Panic behavior disrupted the balance of exit utilization and generated an unbalanced population distribution at each exit, leading to congestion and reduce evacuation efficiency. As seen from Figure 20, the red agent changes its original planned exit and chooses exit V, which is farther away. Therefore, the red-marked agent’s panic behavior will make the VI exit more crowded, while exit V is not fully utilized. The dynamic process similar to the red-marked agent may affect the entire evacuation process, explaining the congestion phenomenon appears again in front of the exit VI in case 04, shown in Figure 21.

Meanwhile, exit V’s overall evacuation time was not as long as exit VI in case 03. However, the ideal result of evacuation is that the two exits could be used reasonably and thoroughly. People can reduce the evacuation time by continuously reducing the distance with others. This contrast proves that all exits were not fully utilized, as shown in Figure 19.

#### 4.3.3. Optimized Evacuation Strategy

A good evacuation strategy can improve the overall efficiency of evacuation. Based on the above results, the exercise organizer should consider the number of participants in the drill and each exit’s actual traffic capacity. A contingency plan for the drill should be formulated so that the proportion of familiar people with each exit is roughly the same as the exit capacity, and it should be released to the public. To avoid the panic effect leading to irrational behavior at the exit, video surveillance equipment, manual intervention, and on-site guidance can be added to control the risk of unreasonable conduct. In the actual evacuation process, the instructor can relieve people’s panic about the ignorance of the exit and improve the exit’s utilization rate and evacuation efficiency. During the drill, evacuation drill training equipment consisting of a series of sensors (such as active RFID) can be equipped to track evacuees. The results of the drill should be analyzed, and the plan was supposed to be optimized to provide a basis for the organization of subsequent evacuation drills.

## 5. Conclusions

Firstly, this paper presents an improved continuum model that could simulate individualized evacuation behaviors. Based on this model, different exit familiarity ratios are set to simulate how trained individuals interact to achieve the goal of evacuating as soon as possible. Firstly, a small simulation based on cellular automata was carried out to verify the micro-level model. It was found that the evacuated training level would affect the evacuees’ choice of exit, thus affecting the escape process. Then, at the macro level, taking a community in Zhuhai, China as an example, the model was used to simulate pedestrians’ dynamic interaction process during the actual scene’s evacuation process. The simulation results show that the maximum evacuation efficiency can be achieved when the proportion of people familiar with each exit is roughly the same as the capacity of each exit. In this case, the final evacuation end time of each exit is almost the same. At the same time, we also discovered another phenomenon. Hesitation in exit choice will reduce the overall evacuation speed and make the area outside the exit more crowded. Two factors have caused this hesitation, one is the judgment of the final waiting time, and the other is the panic mentality caused by the ignorance of the exit. Ultimately, to improve the evacuation efficiency and reduce hesitating behavior during the evacuation, we proposed an evacuation drill strategy. That is, evacuation drills should be carried out in advance. And the commanders should be assigned to guide the crowds in areas where congestion may occur.

However, the model proposed in this article focuses on individual behaviors, such as exit familiarity, visibility, and panic effects. But for a larger scene, such as the evacuation of people in a city, the model’s computational tasks will be quite huge. In the future, the model might be integrated with the macroscopic scale model and can describe the dynamics of the crowds’ emotional state accurately and efficiently. Some tracking equipment should also be equipped to study the actual evacuation behavior of the personnel further.

## Figures and Tables

**Table 1:** The preference groups of the exits divided.

Priority Number	Exit Group	$vis\left(e_{k},r_{i}\right)$	$fam_{i}\left(e_{k}\right)$
1	$E_{i}$(1)	1	1
2	$E_{i}$(2)	0	1
3	$E_{i}$(3)	1	0
4	No Preference	0	0

**Table 2:** The risk factor of each preference groups.

Preference Number	Exit Group	Risk Factor
1	$E_{i}$(1)	1
2	$E_{i}$(2)	1.05
3	$E_{i}$(3)	1.1
4	No Preference	+∞

**Table 3:** The area of emergency shelters selected and the number of evacuees.

Number	Candidate Refuge	Area (m^2^)	Capacity (Number of People)
1	Xiawan Middle School playground	10,879	7252
2	The Second People’s Hospital	3327	2218
3	Zhuhai Nursing School	3100	2066
4	Qiaoguang Primary School	5524	3683
5	Bahui Garden	2579	1720
6	Huaning Greenland Garden	2247	1498
7	Southwest Hotel	2772	1848
8	Open space	2432	1621
9	Hospital parking garage	1983	1322
10	Guangfa Garden grassland	2195	1464

## Data Availability

Data sharing not applicable No new data were created or analyzed in this study. Data sharing is not applicable to this article.
